# The European Medical Device Regulation–What Biomedical Engineers Need to Know

**DOI:** 10.1109/JTEHM.2022.3194415

**Published:** 2022-07-28

**Authors:** Tom Melvin

**Affiliations:** School of MedicineTrinity College Dublin 8809 Dublin 2 D02 VF25 Ireland

**Keywords:** Medical device, regulation

## Abstract

The Medical Device Regulation (EU) 745/2017 (MDR) has replaced the medical device directives which were in place since the early 1990s. MDR introduces a number of changes of relevance to biomedical engineers who work in healthcare institutions or with medical devices. This includes changes relating to devices produced in healthcare institutions, custom-made devices, single use devices, devices without an intended medical purpose, clinical investigations and device traceability. There are also challenges in implementation of the MDR, with a shortage of available notified bodies needed to conduct conformity assessment, with a consequent risk of product unavailability. Understanding these changes is important as implementing new requirements in practice may require additional resources or the introduction of new processes or systems.

## I. Introduction

Biomedical engineering more recently recognised discipline of engineering, [1] and biomedical engineers play an important role in the design, development, integration and management of medical device technologies. Medical devices represent a great diversity of medical technologies ranging from low-risk products such as wound dressings to complex integrated products such as haemodialysis systems.

The European regulation for medical devices has been subject to a significant revision with the Medical Device Regulation (EU) 745/2017 (MDR) [2] replacing the Medical Device Directives (MDD) which had been in force since the 1990s [3]. The MDR is a Regulation, meaning that the legal requirements must be applied equally in all Member States, as opposed to a Directive which allows for some variance in implementation on a national level so long as the overall objectives are met.

The MDR has a transition period for products marketed under the previous rules (the Medical Device Directives) to continue to be marketed until 26 May 2024 at the latest.

**FIGURE 1.**
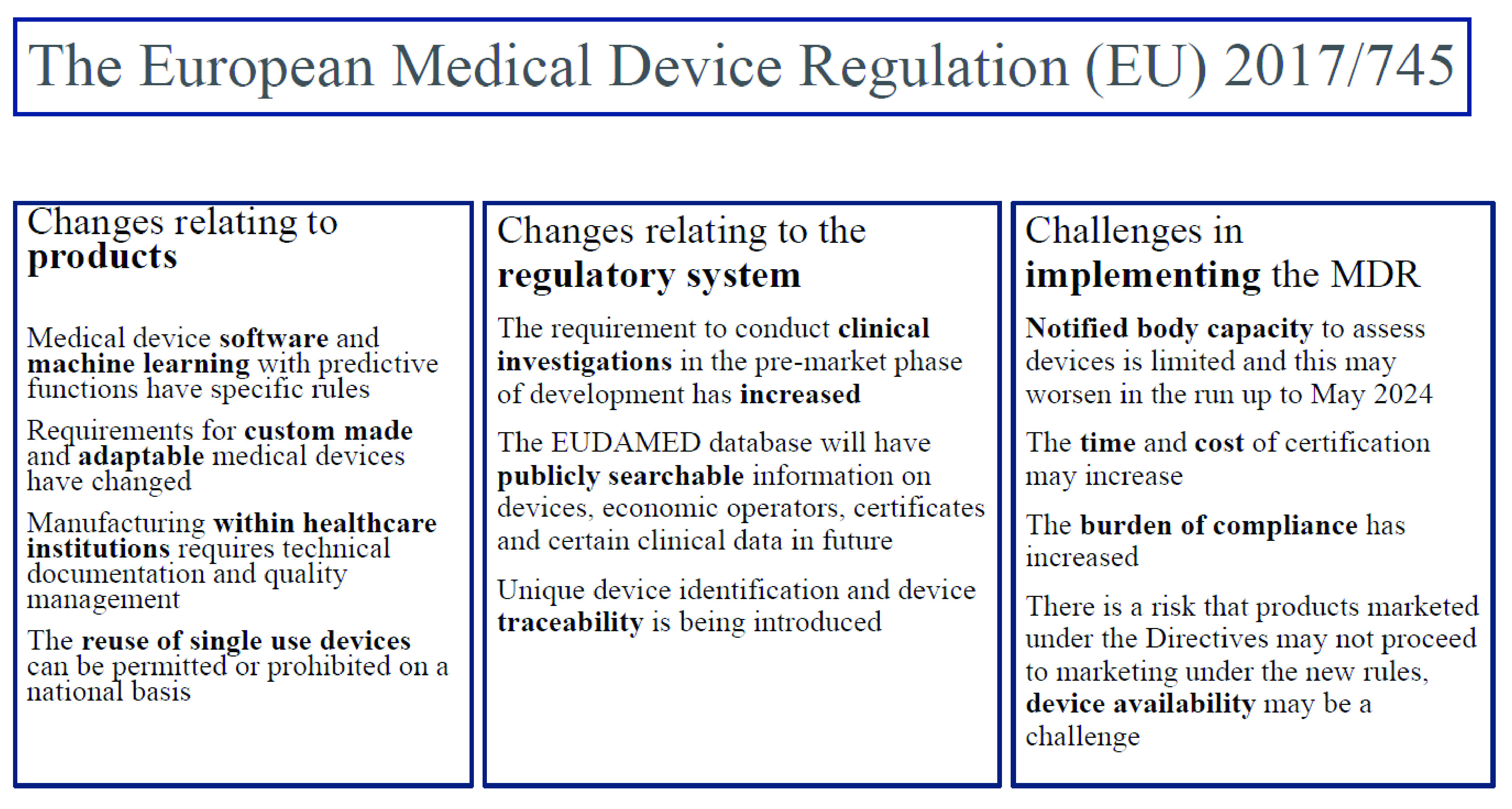
A graphical abstract of changes relevant to biomedical engineers.

In this paper, an introduction to changes in regulation that are of relevance to biomedical engineers are introduced. This paper does not cover legislative changes relating to reimbursement or health technology assessment, [4] *in-vitro* diagnostic products (which have their own revised legislation), [5] or policy work on a national level to translate the MDR into practice. This paper is not an exhaustive demonstration of the requirements that apply, and it is always important to consult the text of MDR to understand the complete requirements which apply. A graphical abstract summarising the changes discussed in this paper is presented in figure 1.

## II. Background to Medical Device Regulation in Europe

When the first regulation was introduced for medical devices in Europe, there was a clear trade and engineering focus in the legislation. At the time, before the MDD took effect, in order to sell a medical device in multiple European Member States, it was necessary to comply with the national standards in each country and as a result of this, product manufacturers were often faced with multiple different sets of test requirements [6]. This created a challenging trade environment within Europe. In order to reduce some of these technical barriers to trade, a type of legislation known as ‘new approach’ legislation was used, which introduced the concept of a single set of essential requirements which applied to all products, and which could be supplemented by the use of standards which when harmonised, allowed a presumption of conformity to those essential requirements.

The use of standards, created by organisations such as the International Standards Organisation (ISO) tended to focus on the processes, testing and manufacturing methods and they have traditionally had an engineering focus, demonstrating for example, the types of bench testing required to assess a family of medical devices.

To market a medical device, a conformity assessment is required. For low-risk devices, (class I) this can be undertaken by the device manufacturer directly, however for higher risk devices, (class IIa, IIb and III) this is required to be conducted by a notified body. Once successfully completed, a CE-mark is awarded, which allows for marketing in all European Member States, without any further technical barriers to trade.

This basic system has been retained with the MDR, albeit with a number of additional processes and requirements [7]. These changes are not disruptive to early-stage medical device development (i.e. conception, prototyping and bench or animal testing), however they introduce a range of important changes with respect to the clinical study of new medical devices, the market entry assessment, and the post-market requirements.

## III. The Scope of the New Regulation

Under the MDD, medical devices were required to have a medical purpose, [8] however this led some products that were similar to marketed medical devices, but which did not have a medical purpose, such as non-corrective contact lenses, to be regulated as general products rather than medical devices.

In order to improve the regulatory oversight of these products, a list of six products has been introduced in Annex XVI of the MDR, including for example, devices used for liposuction, brain stimulators and lasers for uses such as tattoo removal. These products require common specifications to be published in order to allow for the marketing of these products and the European Commission are currently preparing these specifications [9].

Medical device software, certain health apps and machine learning medical devices which utilise predictive functions are now clearly included in the definition of a medical device, [10] and subject to a risk based classification [11]. In addition to compliance with the MDR, in future medical device software utilising machine learning or artificial intelligence may be subject to additional requirements as a result of the proposal for an artificial intelligence regulation in Europe [12]. Other more minor changes in scope to the MDR include rules relating to medical devices which are absorbed by or locally dispersed in the human body [13]. A recently published guidance document helps to determine the borderline between medical device products and pharmaceutical products [14].

## IV. Harmonised Standards and the New Regulation

The use of standards such as those produced by the ISO are a crucially important part of the regulatory system with respect to aspects such as quality management systems, risk management and product testing. As the regulatory system moves from a Directive based system to the MDR, all of the previously harmonised standards have to be reassessed to the new rules. This is a priority for the European Commission, who have engaged harmonisation consultants to undertake the technical work necessary to assess the current standards to the new legislation [15]. A number of standards have been harmonised to the MDR [16]. For standards which have not yet been harmonised, the use of these standards is considered to be ‘state of the art’ and their use is encouraged [17].

## V. New Types of Clinical Investigation Requirements

Under the MDD, an application or notification was required prior to conducting a clinical investigation of a non CE-marked device. Under the MDD there were limited requirements relating to the need to conduct clinical investigations, and the type of clinical investigation that was required. As the MDD contained limited detail, Member States could apply different types of oversight to clinical investigation applications. The MDR has a much larger amount of detail regarding clinical investigations with 20 articles, [17] and information regarding the submission in an Annex [18]. The need to conduct a clinical investigation has also increased, in particular for products classified as high risk, and the ability to claim equivalence as an alternative to conducting a clinical investigation is more limited, with a contract required between manufacturers [19].

The scope of clinical studies which require an application or notification has also changed with the MDR. Post-market clinical investigations, termed ‘PMCF-investigations’ [20] now require a notification and ‘other’ types of clinical investigations not previously covered by the Directives require a notification in accordance with any additional national law [21]. This may affect clinical studies of medical devices which are not conducted by the product manufacturer, but which are conducted for academic purposes. In general, all clinical investigations require an approval by an ethics committee, which are now required to be established under national law [22].

## VI. Rules Regarding Custom-Made Medical Devices

Custom-made medical devices have specific design characteristics which are intended for the sole use of a particular patient and are not generated by mass-manufacturing methods [23]. Custom-made medical devices are very important for certain medical specialisms, for example the use of custom-made implants using 3-D printing techniques. These organisations are in many cases small organisations which may be closely associated with a hospital or clinical practice.

Manufacturers of these products are not required to undertake the typical conformity assessment required for general medical devices, [24] and the general requirements relating to unique device identification, [25] registration of the devices, [26] and preparing a summary of safety and clinical performance, [27] do not apply.

The MDR introduces a requirement to have a person responsible for regulatory compliance (although the necessary qualification is 2 years manufacturing experience, rather than the need for a formal qualification), [28] and to prepare technical documentation and undertake post-market assessment activity [29]. This documentation does not need to be submitted, but has to be maintained in the event that it is requested by a national authority [29].

It is important to note that adaptable medical devices or ‘patient-matched’ medical devices are not considered custom-made medical devices, and they are required to comply with the requirements that apply to general medical devices [30]. As a result of this, some products which may have been considered custom-made under the MDD may now require a notified body assessment and certification.

## VII. In-House Manufacturing of Medical Devices

The production of medical devices within a healthcare institution is also provided for in MDR. Depending on the healthcare institution, this may be of relevance to products such as orthotics or increasingly, it is of relevance to in-house developed software solutions which may meet the definition of a medical device. An example of this would be an in-house developed radiotherapy dose calculator, [31] or a patient management programme which may meet the definition of a medical device, for example by analysing clinical data from patients (for example compliance with a sleep apnoea machine).

The healthcare institutions which make these products are required to document a number of justifications which address aspects such as the following: [32]

• That the medical devices are not produced on an industrial scale,
• The devices meet the specific needs of target patient groups,
• The target patient group’s specific needs cannot be met, or cannot be met at the appropriate level of performance by an equivalent device available on the market.

Health institutions are required to ensure that the manufacture and use of the device are ‘under appropriate quality management systems,’ and the products produced are not allowed to be transferred to another legal entity [32]. In a similar way to custom-made medical devices, the producers of these products are required to prepare technical documentation in the event it is requested by the national authority [32]. The legal responsibilities of manufacturer under the MDR apply to healthcare institutions, so it is important to engage appropriately with the management structure in the institution.

## VIII. The Re-Use of Single Use Devices

Medical devices may be intended for a single use (for example an implanted surgical mesh) or for multiple uses (for example an ultrasound machine). The MDR, for the first time introduced rules relating to the reuse of single use devices [33]. This is a practice which developed in some Member States, as a cost-reduction exercise to allow the reprocessing of medical devices which are intended for a single use, but which could be reprocessed to allow for multiple-uses. The MDR allowed each Member State, on a national basis to introduce legislation to allow for this practice or to prohibit it. There is no centralised listing describing the national policy which has been applied on a Member State level, and therefore it will require some research of the legislation or policy documents in each individual territory.

The MDR noted that the health institution or external reprocessor conducting this activity is required to comply with common specifications, and in their absence with relevant harmonised standards or national provisions in order to ensure an equivalent level of safety and performance to that of the corresponding initial single-use device [33]. The manufacturer of a device may indicate on the labelling that the device can be reused only if it is reconditioned under the responsibility of the manufacturer [34].

## IX. Device Traceability and the EUDAMED Database

The ability to trace the medical devices which patients have had implanted is of fundamental importance to market actions such as field safety corrective actions when a safety issue arises with a medical device. The MDR has introduced rules relating to device traceability by means of unique device identifiers (UDI) which identify the device, UDI device identifier (UDI-DI) and also the lot or batch it was manufactured in, the UDI production identifier (UDI-PI) [35]. For healthcare institutions, there are now requirements to put systems in place to record the identifiers for certain high risk (i.e. class III implantable) medical devices [36].

The use of the UDI to identify information relating to the device in the centralised European database (EUDAMED) is another important development with the MDR, however the EUDAMED database is delayed and will not be available for some time. Until it is available, the European Commission has published a range of alternative technical solutions which are being used until the database is available [37].

In future, the EUDAMED database will be the first centralised database for medical devices in Europe with public searchability for devices, certificates and economic operators [38]. At the moment, there is a voluntary use of a number of completed modules in EUDAMED relating to certificates and economic operators. The EUDAMED system will also improve transparency with respect to clinical evidence with clinical investigation reports and summaries, and a summary of safety and clinical performance (SSCP) being available in future.

## X. Available Guidance for the New Regulation

The Regulation is a detailed legal text, with over 120 Articles and detailed accompanying annexes. To support the implementation of the legal requirements, the European Commission, in conjunction with the Member State authorities (the Medical Device Coordination Group) have published almost 100 guidance documents, called ‘MDCG’ guidance documents [39]. These guidance documents consist of questions and answer documents, templates and policy documents and they can assist when working on particular questions relating to the MDR.

## XI. Current Challenges With Implementation

Implementing the new requirements of the MDR into the sector has given rise to a number of challenges. One of the primary challenges is that of system capacity and publications from an organisation representing notified bodies (TEAM NB) have produced data from their first year of activity under MDR rules which show that the capacity of notified bodies to issue sufficient CE certificates within the transition timeline may not be possible for all products or manufacturers [40]. These capacity challenges may lead to product unavailability in the lead up to May 2024 when the transition period ends, and there may also be challenges for the introduction of innovative medical devices or iterations to currently marketed products.

The medical devices coordination group (MDCG), [41] and the competent authority for medical device group (CAMD), [42] recently issued communications regarding the challenges of notified body capacity. The possible solutions have not yet been described. It is possible that an implementing act may be used to allow the continued marketing of some products certified under the MDD subject to manufacturers demonstrating due-effort in achieving timely certification to MDR.

The MDR does not have any special provisions for therapeutic orphan devices, i.e. devices used in the treatment of rare diseases. These products are characterised by low volume sales and a reduced return on investment. As a result, any change in the time or cost to market leaves these products particularly vulnerable to withdrawal.

In pediatric cardiology for example, the use of adult devices in an off-label way is routine [43]. For example, a renal stent for adult use may be used to treat coarctation of the aorta in a neonate. It follows that a small change in product availability may have significant implications for certain interventions.

Biomedical engineers can support risk mitigation by engaging closely with hospital procurement and clinicians to monitor for changes in product availability. Supporting changes to alternative medical devices and developing strategies such as stockpiling to account for short periods of product unavailability when appropriate may also need to be considered.

## XII. Conclusion

This is a non-exhaustive summary of some of the important changes that the MDR introduces for medical device regulation in Europe. Depending on your healthcare institution and practice, some of these may be more disruptive than others. Ensuring an awareness of these changes within your organisation is important to ensure regulatory compliance.

## Conflict of Interest

The author reports no conflict of interest.

## References

[1] (Aug. 5, 2017). Human Resources for Medical Devices, the Role of Biomedical Engineers. WHO Medical Device Technical Series. Accessed: May 30, 2022. [Online]. Available: https://www.who.int/publications/i/item/9789241565479

[2] Regulation (EU) 2017/745 of the European Parliament and of the Council of 5 April 2017 on Medical Devices, Amending Directive 2001/83/EC, Regulation (EC) No 178/2002 and Regulation (EC) No 1223/2009 and Repealing Council Directives 90/385/EEC and 93/42/EEC.

[3] Council Directives 90/385/EEC and 93/42/EEC.

[4] Regulation (EU) 2021/2282 of the European Parliament and of the Council of 15, Dec. 2021 on Health Technology Assessment and Amending Directive 2011/24/EU.

[5] Regulation (EU) 2017/746 of the European Parliament and of the Council of 5, Apr. 2017 on In Vitro Diagnostic Medical Devices and Repealing Directive 98/79/EC and Commission Decision 2010/227/EU.

[6] J. Pelkmans, “The new approach to technical harmonization and standardization,” J. Common Market Studies, vol. 25, no. 3, p. 249, Mar. 1987.

[7] European Commission, Public Health, Medical Devices, Sector. Accessed: May 30, 2022. [Online]. Available: https://ec.europa.eu/health/medical-devices-sector/new-regulations_en

[8] Article 1(2), Council Directive 93/42/EEC.

[9] European Commission, Manufacturers of Devices Without an Intended Medical Purpose. Accessed: May 30, 2022. [Online]. Available: https://ec.europa.eu/health/medical-devices-new-regulations/getting-ready-new-regulations/manufacturers-devices-without-intended-medical-purpose_en

[10] Article 2(1), Second Indent, MDR.

[11] Annex VIII, Rule 11, MDR.

[12] Proposal for a Regulation of the European Parliament and of the Council Laying Down Harmonised Rules on Artificial Intelligence (Artificial Intelligence Act) and Amending Certain Union Legislative Acts, Document 52021PC0206, COM/2021/206 Final. Accessed: Jul. 14, 2022. [Online]. Available: https://eur-lex.europa.eu/legal-content/EN/TXT/?uri=CELEX%3A52021PC0206

[13] Article 52(11), MDR.

[14] (Apr. 2022). Medical Device Coordination Group Document MDCG 2022–5, Guidance on Borderline Between Medical Devices and Medicinal Products Under Regulation (EU) 2017/745 on Medical Devices. Accessed: Jul. 14, 2022. [Online]. Available: https://ec.europa.eu/health/system/files/2022-04/mdcg_2022-5_en_0.pdf

[15] (Apr. 2021). Medical Device Coordination Group Document MDCG 2021–5, Guidance on Standardisation for Medical Devices. Accessed: May 30, 2022. [Online]. Available: https://ec.europa.eu/health/system/files/2021-04/md_mdcg_2021_5_en_0.pdf

[16] European Commission, Regulation (EU) 2017/745 on Medical Devices—Summary List as Pdf Document. Accessed: May 30, 2022. [Online]. Available: https://ec.europa.eu/docsroom/documents/50115

[17] Articles 62 to 82 of MDR.

[18] Annex XV, MDR.

[19] Article 61(5), MDR.

[20] Article 74, MDR.

[21] Article 82, MDR.

[22] Article 62(3), MDR.

[23] Article 2(3), MDR.

[24] Article 10(6), MDR.

[25] Article 27(1), MDR.

[26] Article 29, MDR.

[27] Article 32(1), MDR.

[28] Article 15(1), MDR.

[29] Annex XIII, MDR.

[30] (Mar. 2021). Medical Device Coordination Group Document MDCG 2021–3, Questions and Answers on Custom-Made Devices & Considerations on Adaptable Medical Devices and Patient-Matched Medical Devices. Accessed: May 30, 2022. [Online]. Available: https://ec.europa.eu/health/system/files/2021-03/mdcg_2021-3_en_0.pdf

[31] (Oct. 2019). Medical Device Coordination Group Document MDCG 2019–11, Guidance on Qualification and Classification of Software in Regulation (EU) 2017/745—MDR and Regulation (EU) 2017/746—IVDR. Accessed: May 30, 2022. [Online]. Available: https://ec.europa.eu/health/system/files/2020-09/md_mdcg_2019_11_guidance_qualification_classification_software_en_0.pdf

[32] Article 5(5).

[33] Article 17, MDR and Preamble 38, MDR.

[34] Annex I, section 23 (4)(o), MDR.

[35] Article 27(1), MDR.

[36] Article 27(9), MDR.

[37] (May 2021). Medical Device Coordination Group Document MDCG 2021–1 Rev.1 Guidance on Harmonised Administrative Practices and Alternative Technical Solutions Until EUDAMED is Fully Functional. Accessed: May 30, 2022. [Online]. Available: https://ec.europa.eu/health/system/files/2021-05/2021-1_guidance-administrative-practices_en_0.pdf

[38] European Commission. EUDAMED—European Database on Medical Devices. Webpage. Accessed: Jul. 14, 2022. [Online]. Available: https://ec.europa.eu/tools/eudamed/#/screen/home

[39] European Commission. Guidance—MDCG Endorsed Documents and Other Guidance. Accessed: May 30, 2022. [Online]. Available: https://ec.europa.eu/health/medical-devices-sector/new-regulations/guidance-mdcg-endorsed-documents-and-other-guidance_en

[40] The European Association of Medical devices Notified Bodies Medical Device Survey 2021 Data From All Members (End 2021). Accessed: May 26, 2022. [Online]. Available: https://www.team-nb.org/wp-content/uploads/2022/05/Survey-2021-20220516-1.pdf

[41] (Jun. 2022). Medical Device Coordination Group Document, MDCG 2022-11, MDCG Position Paper: Notice to Manufacturers to Ensure Timely Compliance With MDR Requirements. Accessed: Jul. 14, 2022. [Online]. Available: https://health.ec.europa.eu/latest-updates/mdcg-2022-11-mdcg-position-paper-notice-manufacturers-ensure-timely-compliance-mdr-requirements-2022-06-13_en

[42] (Jun. 2022). Competent Authority for Medical Devices, CAMD Statement—50th CAMD Plenary Meeting. Accessed: Jul. 14, 2022. [Online]. Available: https://www.camd-europe.eu/events/camd-statement-50th-camd-plenary-meeting/

[43] J. S. Sutherell, R. Hirsch, and R. H. Beekman, “Pediatric interventional cardiology in the United States is dependent on the off-label use of medical devices,” Congenital Heart Disease, vol. 5, no. 1, pp. 2–7, Jan. 2010, doi: 10.1111/j.1747-0803.2009.00364.x. 20136851

